# Retraction Note: Candesartan Protects Against Cadmium-Induced Hepatorenal Syndrome by Affecting Nrf2, NF-κB, Bax/Bcl-2/Cyt-C, and Ang II/Ang 1–7 Signals

**DOI:** 10.1007/s12011-024-04347-6

**Published:** 2024-08-15

**Authors:** Esam O. Kamel, Wail M. Gad-Elrab, Mohammed A. Ahmed, Zuhair M. Mohammedsaleh, Emad H. M. Hassanein, Fares E. M. Ali

**Affiliations:** 1 Department of Medical Histology and Cell Biology, Faculty of Medicine, Al-Azhar University, Assiut, Egypt; 2 Department of Human Anatomy & Embryology, Faculty of Medicine, Al-Azhar University, Assiut, Egypt; 3 Pathology Department, Faculty of Medicine, Al-Azhar University, Assiut, Egypt; 4 Department of Medical Laboratory Technology, Faculty of Applied Medical Sciences, University of Tabuk, Tabuk, 71491 Kingdom of Saudi Arabia; 5 Department of Pharmacology and Toxicology, Faculty of Pharmacy, Al-Azhar University, Assiut Branch, Assiut, 71524 Egypt

**Retraction Note: Biological Trace Element Research (2022) 201:1846–1863**

10.1007/s12011-022-03286-4

The Editors-in-Chief have retracted this article. After publication, concerns were raised regarding some of the images presented in the figures, specifically:

- Fig. 3B and D appear to overlap;
- Fig. 7A and D appear to overlap;
- Figs. 10C and 13B appear to overlap.

Due to the number of concerns, the Editors-in-Chief no longer have confidence in the presented data.

None of the authors agree to this retraction.

